# Covid-19 and school closure: Examining the impact on private mid-range and low-fee private basic schools in Ghana

**DOI:** 10.1007/s11125-021-09579-1

**Published:** 2021-12-20

**Authors:** Lordina Juvenile Ehwi, Richmond Juvenile Ehwi

**Affiliations:** 1 Cambridge Assessment International Education, Cambridge University Press and Assessment, The Triangle Building, Shaftesbury Rd., Cambridge, CB2 8EA UK; 2 Department of Land Economy, Cambridge Centre for Housing and Planning Research, University of Cambridge, 19 Silver Street, Cambridge, CB3 9EP UK

**Keywords:** Covid-19, Private schools, Organizational ambidexterity, Greater Accra Metropolitan Area, Ghana

## Abstract

The Covid-19 lockdown implemented globally to prevent the spread of the virus has led to the closure of schools. However, insight into the impact of the lockdown on private schools and the responses it has elicited is limited, especially across the African continent. This article examines the impact of the lockdown on private basic schools in Ghana and how they responded to the closure. Following “organizational ambidexterity” and qualitative interviews with nine proprietors of private schools in Ghana, the study found that the schools’ closure had a negative impact on private basic schools in five crucial ways: disruption to teaching and learning, difficulty in retrieving unpaid teaching fees, inability to pay staff salaries and statutory payments, underutilization of existing assets, and the cost of storing unused stock. The article offers suggestions to the government to support private schools that are broadening educational access at thin profit margins.

In March 2020, the novel coronavirus was declared a global pandemic by the World Health Organization (WHO, 2020) and national governments worldwide introduced various lockdown measures. Among these was the closure of schools, interrupting *traditional schooling* (i.e., face-to-face teaching in which students and teachers are physically present in a classroom, giving and receiving tuition) for 1.5 billion pupils worldwide for at least the 5 months following the WHO declaration (UNESCO, 2020). In Ghana, all educational institutions, both private and public, were also asked to close on March 16, 2020, following partial lockdown in the Greater Accra Metropolitan Area (GAMA) and the Greater Kumasi Metropolitan Area (GKMA)—the largest urban conurbations in Ghana. Although the lockdown was lifted after only 3 weeks, pre-tertiary schools remained partially closed and were expected to resume fully in January 2021 (Akuffo-Addo, 2020). All pre-tertiary schools, comprising preschool, primary, junior secondary and senior secondary, were closed, and students sent home. Only those in the penultimate year and final year were allowed to attend school.

In Ghana, as elsewhere, the lockdown affected various stakeholders, especially pupils, teachers, and school proprietors. For example, it was reported that school closures adversely affected 9.2 million learners (Ministry of Education, 2020). While the literature on Covid-19 and its impact on schools is growing, limited attention has been paid to the impact of school closures on private schools in terms of how the lockdown affected them and how they responded. Although Niazi and Doorly (2020) recently published a report that explored the impact of Covid-19 on the non-state education sector in six low-and middle-income countries (i.e., India, Pakistan, Nigeria, Ghana, Kenya, and Colombia), it is unclear how many school proprietors were interviewed in each country, and the report did not make clear the current and future coping strategies schools adopted. Thus, a gap exists in our understanding of the impact of Covid-19 on schools, and their current and future coping strategies. In this article, we focus on the impact of the closure on private basic schools, mainly because the majority of these schools have a sole proprietor, receive no government subvention, and hence rely on tuition fees from parents to meet both operational and statutory expenses (Inusah, 2020). Yet, private schools remain key players in delivering quality education in the educational ecosystems of most developing countries (Baum et al., 2018; Inusah, 2020). Therefore, it is crucial to understand how the closure of schools affected these schools and how they responded to their mandate of broadening access and improving quality education. Against this backdrop, we formulated three questions: (a) What was the impact of the school closures on private basic schools? (b) How did schools respond to the impact of school closures? (c) What organizational and management factors enabled schools to respond to the school closures?

Answering these questions yields useful insights for policy makers to fashion policies to better support private schools, given their critical role in broadening educational access (Niazi & Doorly, 2020) and offering better learning outcomes (Day Ashley et al., 2020; Ohara, 2013). Also, the insights could help private schools reposition themselves to become more resilient during future pandemics.

In the bourgeoning literature on the Covid-19 lockdown and schooling (e.g., Azevedo et al., 2021; Brom et al., 2020; Cahapay, 2020; Castro, 2020; Popyk, 2020; Power, 2020; Williamson et al., 2020), we found limited insights into how the closure of schools affected private schools and how they responded to school closures. Also, insights from African countries remain scarce. Although a rapid-review report on the impact of Covid-19 on non-state schools in six countries across three continents by Niazi and Doorly (2020) reported findings consistent with some of the challenges of school closure discussed here, it lacked empirical data to substantiate claims made regarding the non-payment of teachers’ salaries and the online challenges facing schools. Hence, an empirical study into the impact of school closures on private basic schools and the responses this elicited is warranted.

## The private school landscape in Ghana: Typologies and characteristics

Schools at the pre-tertiary level in Ghana bifurcate into public and private schools at both the secondary and basic school level (i.e., senior high schools [including vocational and purely technical schools] and basic schools). Public senior high schools comprise schools established by the state under the Ghana Education Trust Fund and those that were established by missionaries and are now either fully or partially run by the state. They constitute the majority of secondary schools in Ghana and now charge no tuition fees, owing to the "free SHS" (senior high school) policy implemented by the New Patriotic Party (NPP; Free SHS Ghana, 2018). Public basic schools comprise schools built and operated by district assemblies and those established by local communities but operated by district assemblies. Furthermore, no tuition fees are charged in public basic schools (Akyeampong & Rollestone, 2013).

**Figure 1:**
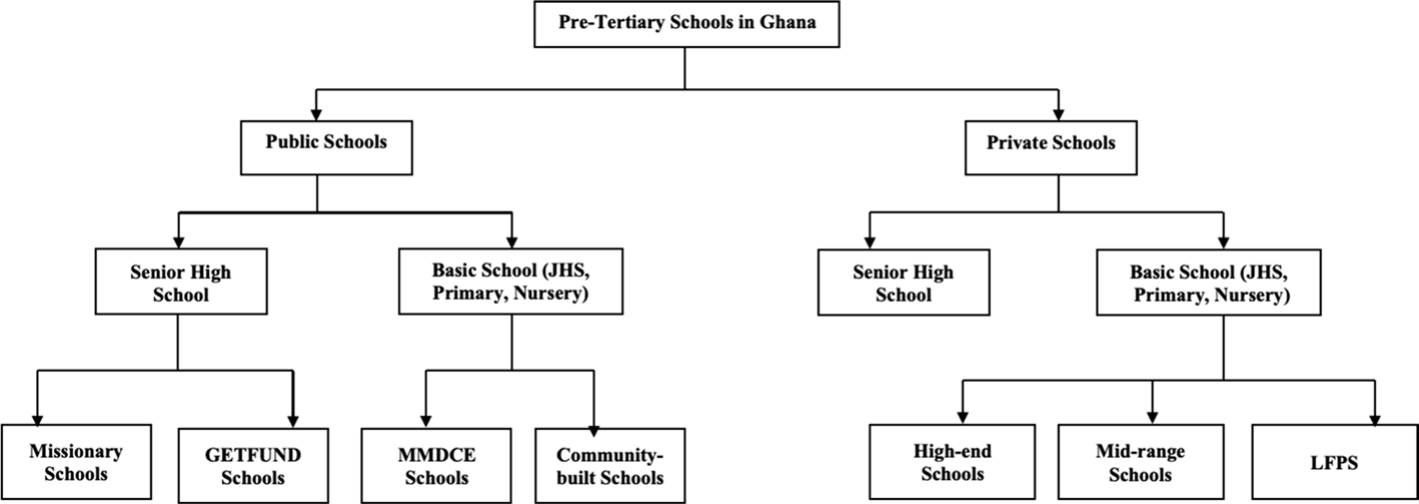
The ecosystem of pre-tertiary schools in Ghana. *Source:* Authors’ construct

Both private senior and basic high schools, on the other hand, comprise schools built and operated by private individuals or companies. These schools charge fees for tuition and other logistics and hence are perceived to be profit-making organizations as the 1992 constitution of Ghana allows anyone acting within the remits of the law to establish a private school (Republic of Ghana, 1992). At the basic level, private schools are distinguished based on how high the tuition fees they charge are, the caliber of teachers they attract, and the assets and physical infrastructure they own. Based on these matrices, private schools are usually classified as high-end, mid-range, and low-fee private schools (LFPS; Baum et al., 2018; Mcloughlin, 2013) (Figure 1).

High-end private schools attract children from elite households and have considerable assets, such as fleets of buses, state-of-the-art teaching, learning facilities (e.g., well-resourced information communication technology [ICT] centers with Internet connectivity), etc. They recruit qualified international and local teachers. They are often located in high-income residential neighbourhoods and charge exorbitant fees, sometimes priced in US dollars (see, for example, Tema International School, 2020).

Mid-range private schools are mainly indigenously owned and target children from lower- and upper-middle-income households, such as those of journalists, bankers, and university lecturers. They usually have moderate assets, such as a couple of buses, and modest infrastructure, such as ICT laboratories and libraries. They also recruit trained teachers, university and polytechnic graduates, and sometimes even brilliant senior high school leavers. Their fees are lower than those of high-end schools but are still expensive for low-income households. They tend to be more geographically diverse than high-end private schools (see, for example, the Desk Preparatory School website).

LFPSs were set up to offer children from low-income and disadvantaged households the opportunity to receive arguably higher quality education than that offered by public basic schools. They comprise international chain schools, philanthropically supported schools, and independent or sole proprietorship schools (Mcloughlin, 2013). With notable exceptions (i.e., those belonging to the international chain LFPSs and philanthropic LFPSs set up with external international support), the majority of LFPSs, especially independent LFPSs, are set up by sole proprietors. Owing to their limited capital base, they usually provide basic infrastructure, such as classrooms, toilets, and a canteen (Akaguri, 2011). Most lack libraries, computer laboratories, and staff common rooms (Ohara, 2013). The low tuition fees charged mean they are unable to recruit highly trained teachers. Hence, they are often compelled to hire unemployed university and senior high school graduates. These schools tend to have higher staff attrition rates and mainly rely on fees for daily operation (Adelabu & Rose, 2004; Ohara, 2013; Srivastsava, 2013).

Given the considerably differences in the private basic schools (outlined above), it is reasonable to expect the impact of school closure and the strategies adopted to respond to it will vary. Hence, in this article we empirically explore the impact of school closure on private basic schools and the strategies such schools adopted during school closures.

## Theoretical framework and methodology

### Organizational ambidexterity

Organizational ambidexterity is an adaptive system (Greve, 2007) that offers insights into how organizations respond to changes (Shoba, 2017; Tushman & O’Reilly, 1996). Organizational response to changes include purely exploitative and explorative strategies or some combination of both (Zhou et al., 2016). Exploitative means organizations are taking advantage of the assets and resources at the disposal to respond to changes while explorative imply that organizations look for new ways, and resources to respond to changes (Birkinshaw & Gibson, 2004). How far an organization will go in using either exploitative or explorative strategies or both depend on environmental, Organizational and management factors (Lavie et al., 2010). Environmental factors draw attention to issues such as general market competition and exogenous shocks (e.g., introduction of regulations and appropriability regimes), which allow organizations to innovate, protect their innovation and profit from their inventions.

Organizational factors focus on issues related to excess assets, human resource capacity, and logistics, which organizations can draw upon to respond to changes (Voss et al., 2008). These factors also draw attention to issues regarding Organizational structure, culture, identity, size, and age, which all together influence what is permissible in an organization, how things can and should be done, and how quickly they can be done (Lavie et al., 2010).

Finally, managerial factors such as attitudes toward risk, how feedback on performance are given, and the past experiences of senior management during turbulent times all influence whether an organization will adopt either an exploitative or explorative strategy, or both.

### Conceptualizing the impacts and response of Covid-19 schools’ closure on private schools in Ghana

**Figure 2:**
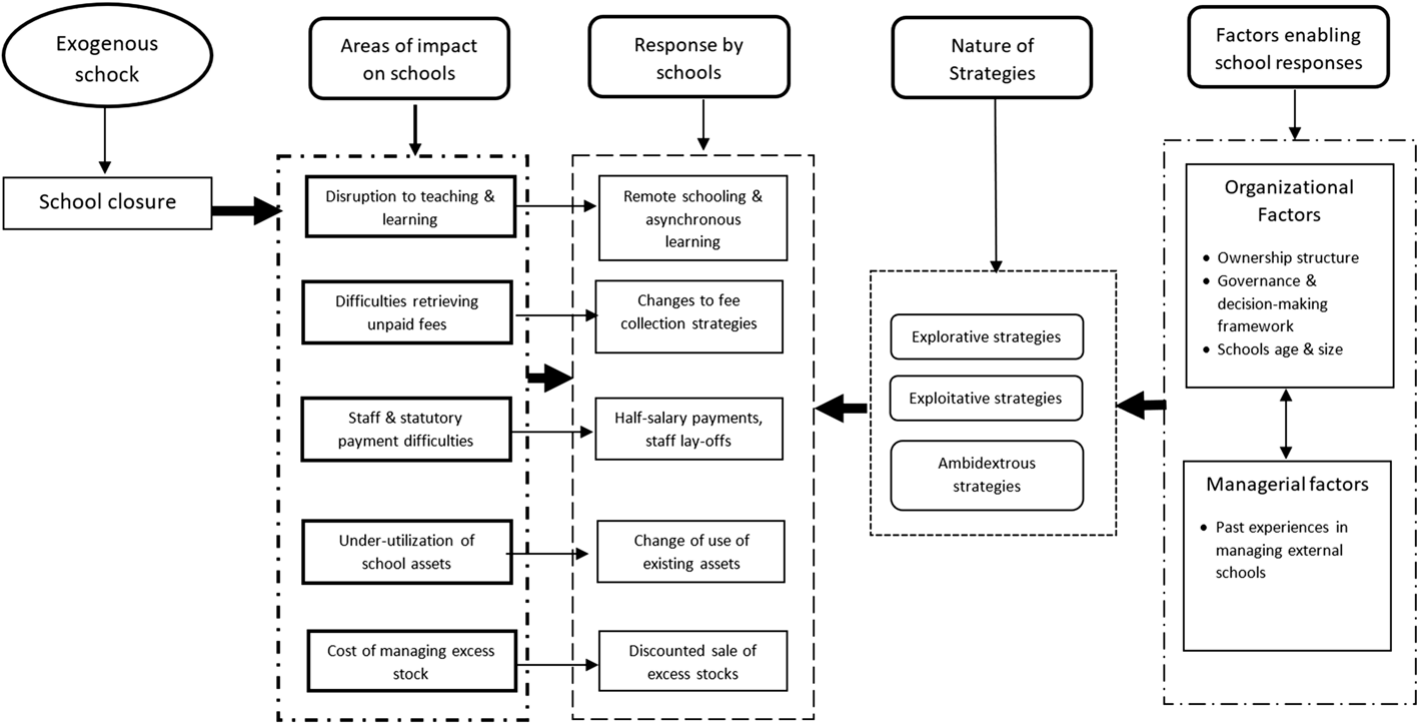
Conceptual framework showing the connection between Covid-19-induced school closures, impacts, responses, and enabling strategies. *Source:* Authors

In this article, we draw on some of the concepts from organizational ambidexterity elaborated above to explore how private basic schools in Ghana have responded to the impacts of school closure and the factors that have enabled their responses. To do so, we first conceptualize the closure of schools in Ghana as an exogenous shock (see Lavie et al., 2010) that was unanticipated by the school management. This exogenous shock, we contend, is likely to have affected several aspects of private schools, including but not limited to the organization of teaching and learning, retrieval of unpaid tuition fees, payment of staff salaries and statutory fees, underutilization of school assets, and the cost of unused stock. These impacts are likely to have compelled schools to take responsive actions underpinned by explorative, exploitative, or ambidextrous strategies aimed at addressing the impacts of school closures. We further contend that the response strategies adopted by schools would be facilitated by organizational factors, such as ownership structures and decision-making frameworks; individual school missions; age and size of the schools; and managerial factors, such as past experiences of school management in dealing with exogenous shocks. This conceptual framework is summarized in Figure 2. We draw on this framework to guide both the presentation of our empirical findings and the discussion.

### Methodology

The study adopted a qualitative method informed by an interpretivist ontology (Creswell, 2018). We drew empirical data from nine basic private school proprietors within the GAMA who had privileged information about how their schools were affected by the pandemic. GAMA was chosen because it was one of two metropolitan areas in Ghana that went under total lockdown from March 16 to April 19, 2020.

The study adopted a purposive sampling approach, beginning with a snowball of school proprietors with whom both authors had established acquaintance. This sampling approach was deemed suitable because the time of the data collection (April and May 2020) coincided with when most school proprietors and head teachers were still exploring how they would support final-year students preparing to sit for their final examinations under the Covid-19 restrictions. Hence, many turned down our invitation for an interview. However, as the study was exploratory and sought to gain in-depth and context mediated insights from respondents with strategic and operational responsibilities (Bryman, 2012), “meaning and coding saturation,” rather than a large sample size, were the crucial determinants of sample adequacy (Hennink et al., 2017).

The data were collected using semi-structured telephone interviews. Each interview lasted approximately 25 minutes, and all were recorded with the interviewee’s consent. The audio files were subsequently transcribed using the software Otter.ai. The transcripts were analyzed using simple thematic analysis involving separate extraction of themes related to the impact of the school closure and strategies adopted by each author. The authors later exchanged the themes each had written down, reviewed them, selected common themes, and made a decision regarding the inclusion of uncommon themes (Attride-Stirling, 2001). Notable quotes that illuminated the themes identified were included in the text after they had been anonymized using the following acronyms: SC for school and SCP for school proprietor. To avoid confusion, all head teachers were also referenced as SCP.

## Findings

### The demography of respondents and their schools

Eight out of nine interviewees described themselves as sole proprietors; one person said they jointly owned the school with their spouse. The nine interviewees comprised four females and five males. The age range for the females was 40 to 58 years, and that of the males was 38 to 55 years. Four out of nine interviewees possessed a university degree, while the remaining five had higher national diplomas. Although the interviewees appeared to be highly educated, none had received formal training and certification in educational leadership and school management.

Regarding the schools represented, six schools (75%) were LFPSs, while the remaining two (25%) were mid-range private schools. It was evident that the mid-range schools charged higher tuition fees than did the LFPSs, the lower and upper limits of both school types being Gh¢500 to Gh¢1,000 and Gh¢150 to Gh¢700, respectively (Table 1). The average student population for the nine schools was 416. The average number of staff was estimated to be approximately 19, except for SC4, which had 24 staff members, and SC6, which had five staff members. The staff to student ratio was 1 staff to a modest 25 students.

**Table 1:** Summary information about schools selected. *Source:* Authors’ field data (April 2020). *Note:* TMA – Tema Metropolitan Assembly, LEKMA – Ledzokuku-Krowor Municipal Assembly.

School code	District	Type	Averaged fees charged (in Ghc)	Student population	Staff population	Staff:Student ratio	Years in operation	% of students from disadvantaged background
SC1	TMA	Mid-range	500–800	500	14	1:36	19	10–15
SC2	LEKMA	Mid-range	750–1000	800	18	1:44	22	5
SC3	Tema West	LEPS	300–360	300	10	1:30	10	–
SC4	LEKMA	LEPS	360–450	300	24	1:13	12	20
SC5	LEKMA	LEPS	345–700	352	15	1:23	18	30
SC6	TMA	Mid-range	900	30	5	1:6	9	30
SC7	Adentan	LEPS	150	573	37	1:15	13	2
SC8	Tema West	LEPS	350–600	190	13	1:15	19	80
SC9	Tema West	LEPS	300–500	280	15	1:19	20	70

Furthermore, considerable variation existed among the schools. On average, the schools considered had been in operation for nearly 18 years. Five schools had been in existence for more than 15 years, while the remaining four had been operating for less than 15 years. In terms of the proportion of students from financially poor homes offered a place, only in two schools (SC7 and SC9) was this figure higher than 50%. In three schools, between 20% and 30% of the student population came from poor backgrounds. The findings seem to suggest that LFPSs that charge relatively lower tuition fees offer more access to students from poor backgrounds than do mid-range schools that charge relatively higher tuition fees. We now turn to the impact the Covid-19 schools’ closure had on the selected schools.

### The impact of Covid-19 school closures on selected schools

#### Disruption to teaching and learning

Typically, teaching and learning activities in pre-tertiary schools in Ghana are designed to take place within the three academic terms of one year (Little, 2010). These activities, including the organization of end-of-term examinations, usually take place face-to-face. Interviewees said the government’s directive that all schools close came about 3 weeks before the end of the second term (January to April). This period, according to the proprietors, was crucial because the week before the start of exams is usually the revision week and teachers use this time to go over materials they have delivered during the term and answer lingering questions from students. Thus, the unexpected nature of the announcement meant the revisions could not occur in the traditional sense, as the following quote from an interviewee indicates: “We were getting ready for the second terms exams and suddenly we heard from government that all schools must shut. So we couldn’t do revision” (SCP 9).

According to some proprietors, although the government’s directive permitted final-year students preparing to take their Basic Education Certificate Examination (BECE) to attend school and have classes in a socially distanced manner, some parents kept their children out of the school due to uncertainties and fear their children might catch the virus. One proprietor observed:

We noted that some of the final years were not showing up, so we followed up on them and their parents said, they will be learning at home, so we went ahead with the exam preparation for those who were present. (SCP 9)

Taken together, disruptions to teaching and learning had a disproportionate impact on students at lower levels of basic education (i.e., from kindergarten to junior high school [JHS] 2) than on those in the final year (JHS 3), as the latter’s impending exams made them the priority in every school.

#### Difficulty retrieving unpaid tuition fees

The difficulty retrieving unpaid tuition fees was another area in which school proprietors felt the impact of school closures. In most LFPSs and some mid-range private schools, it is typical for the management to allow parents who cannot make advance payment of their wards’ tuition fees to do so in installments. According to some proprietors, these payment arrangements are consonant with their motivation to broaden educational access and improve educational quality for all children (Day Ashley et al., 2020; Tooley, 2009). The implication of this arrangement is that most LFPSs and mid-range schools operate on thin profit margins. Against this backdrop, the suddenness of the lockdown announcement, coupled with this flexible system, made it difficult for the schools to retrieve tuition fees, some being compelled to fall back on their (limited) reserves to keep their schools running. The quote below is illustrative:

Now, the challenge we had initially was that we had not collected all our fees yet. And so, as we must go home. The question is how to get the rest of your money? Like I said, we have a mixture of parents, so a few that sent it to us via the bank, but majority too have not sent. Some of them have already also paid half. (SC1P1)

It is worth noting that, in addition to tuition fees, some schools charged feeding and examination fees. The former caters (for) the meals offered to students, while the latter pays for stationary, printing, and sometimes exam invigilation costs. According to two proprietors (SC9P9 and SC8P8), their schools sometimes prefinanced the preparation of school meals, under the impression that the cost would be recovered through the feeding fees charged every working day or at the start of each week. Thus, as with tuition fees, this additional income was unlikely to be paid by parents, some of whom did not want to pay tuition fees.

#### The inability to pay staff salaries and statutory obligations

According to school proprietors, they are mandated by law to pay their staff’s social security contributions, in addition to their monthly salaries. Furthermore, they have other financial obligations, including the payment of property rates, business operating licenses, and utility bills (e.g., water and electricity) that must be paid irrespective of whether school is in operation or not. Consistent with observations in the literature (Day Ashley et al., 2020), all the proprietors admitted that these financial obligations were mainly financed from tuition fees (Inusah, 2020). Thus, the huge amount of tuition fees in arrears meant some schools risked defaulting on these statutory payments. This is what some proprietors said about the how the lockdown affected their ability to pay salaries:

Given the amount of school fees in arrears, we will not be able to pay the teachers their full salary for March. As for April and the remaining months, we don’t know what will happen. (SC9P9)

The finances are a challenge because we have a payroll of about GH¢60,000. We are just falling on our reserves now and we hope this does not continue for long, otherwise it will impact our operations. (SC2P2)

These assertions are consonant with findings uncovered in Niazi and Doorly’s (2020) study: “Over 50% of schools’ owners have uncollected fees between US$5,600 and US$6,720” (p. 9). When asked whether they had received any support from the government in terms of tax holidays or from private school associations as local businesses, the answer was an emphatic no, although some proprietors indicated they had heard rumors that the government had put in place support packages that were being distributed by the association of private schools. However, for proprietors, the possibility of benefiting from this was remote for several reasons:

In terms of the association, I do not think I am a member, because I attended their meeting twice but did not see any benefit. Also, they are asking us to pay GH¢333.00 to join, so that when the government brings out a business stimulus package, we can get some. However, in these financial times, I do not know why they are asking for that amount. (SC2P2)

I even had a message that the government was organizing an incentive to try and cushion the schools. Then it came on the GES Tema WhatsApp page, but when you look at the criteria, I left it. Yes, because they were asking for the SSNIT [payment record]. In our school, we have only four professional teachers—me, my wife, and two others. We cannot employ trained teachers, because we cannot pay them. The pupil teachers just join for a short time and then they leave, so it did not make sense to register them for the SSNIT. So we decided not to go ahead with the application for the stimulus package, because providing the SSNIT of all the staff members was a prerequisite. (SC3P3)

These quotes reveal how the staff recruitment policy of schools, combined with difficulties in executing statutory obligations, precluded them from receiving government stimulus packages.

#### The underutilization of existing assets

Aside from the classroom blocks and other building stock, such as libraries and computer laboratories, some schools own assets, such as school buses and stationary shops, that complement school activities. For example, apart from transporting students, school buses are used to convey groceries and other foodstuffs procured by the school. According to some proprietors, during vacation periods, classroom blocks are used for organizing vacation classes. Thus, throughout the year, most school assets are used intensely, yielding in some cases extra income. However, because of the school closures, school proprietors said their classrooms would be underutilized for a considerable part of the year and this would cost them money:

We would have been on vacation in 3 weeks’ time, and vacation classes would have started the week after. But because of this lockdown,… this year we cannot organize our vacation classes, and you know that is money we are going to lose. (SC9P9)

Another proprietor, whose school had rented a shop a few blocks from the school to sell stationary, said, “Student footfall to the shop will fall because of the lockdown, and we still have to pay rent for the shop” (SC8P8). This suggests that the underutilization of existing school assets results in the loss of secondary sources of income to schools.

#### The cost of storing unused stock

As part of effective resource management, some schools procure foodstuffs and ingredients in bulk, such as frozen fish and chicken, bags of dry corn, and wheat, for storage and later use. According to proprietors, they make these procurements on the assumption that school activities will continue, and these foodstuffs will be used within a specified period. Unfortunately, given the unanticipated nature of the school closures, some proprietors said they still had unused stock that was becoming costly to store. One proprietor said, “In my school, every month we buy Gh¢2,500 worth of frozen chicken and 16 bags of dry corn. Now our capital is locked up because of the lockdown” (SC9P9). Some proprietors, hopeful that the lockdown would ease soon, kept these stocks of foodstuffs, sometimes at great cost due to the high electricity bills they had to pay.

The next section presents findings relating to how the schools responded to the impacts discussed above.

### Schools’ responses to the impact of the school closures

As Lavie et al. (2010) observed, during exogenous shocks, such as pandemics, organizations adopt either explorative or exploitative strategies or some combination of both, to respond to the impact of the shocks to their organizations. The following section describes responses schools adopted to deal with the impact of the closures discussed.

#### Remote schooling and asynchronous learning

When schools were closed, there were widespread concerns that learning would significantly fall behind without some form of remote learning, especially in developing countries (Azevedo et al., 2021). In Ghana, the government showed leadership in this regard in that, within one month of the school closures, the Ghana Education Service (GES) published a report detailing the risks associated with the disruption to schooling and recommended three major media lead remote learning (Ministry of Education, 2020). These were television, radio, and the Internet. Final-year students in senior and junior high schools who were preparing for their examinations could access a specially designed electronic learning platform by logging in with their student index number (National Inspectorate Board, 2020). Also, radio lessons were designed and launched on June 15, 2020, through a partnership involving the Ghana Broadcasting Corporation (GBC) and affiliate stations in 10 out of the 16 regions. Lessons were aired for 30 minutes once a day and repeated on Tuesdays, Thursdays, and Saturdays (GES, 2020).

Interviews revealed that most school proprietors welcomed this government intervention primarily because it enabled some form of teaching and learning, without the schools incurring any (significant) costs, except those related to sending the online school timetable and reminders to parents. This was summed up by one proprietor as follows:

Now we are doing something on television daily, the government has brought something that’s called “TV learning”. So what we do is that we have given some of the parents the timetable, where they can follow and then assist their children. So when they find any difficulty, they bring it to us. (SC4P4)

Alongside government intervention, some schools took the initiative to facilitate some form of remote learning. This involved using social media platforms, chiefly WhatsApp, to both send and receive examination questions and assignments. According to the proprietors, this strategy took advantage of the widespread use of mobile phones and the ubiquity of the WhatsApp mobile application in Ghana. This is how one proprietor captured their efforts to move toward online schooling: “The school has created a WhatsApp group for the parents and uses this platform to regularly send assignments and exercises to students. When the students complete their assignments, they take pictures and send them back to us” (SC8P8).

It should he emphasized that, while the ubiquity of the WhatsApp application made it easy for schools to send assignments to students in the form of picture messages, this approach was likely to be problematic for two reasons. First, its effectiveness depended on parents having smart phones with Internet connectivity to download the assignments. Thus, for students whose parents do not own smart phones or cannot download data when the assignments are released, this can potentially lead to failing to submit the assignment on time. Second, given that most phones belonged to and were in the custody of the parents, there was a danger of other uses for the phone (e.g., interference from incoming calls) when students were using the phone for learning. Some proprietors admitted to such problems with online schooling.

It is also worth mentioning that not all schools were able to adopt online schooling. For some proprietors, reasons for this included the economic impoverishment of some parents, low levels of digital literacy, and parents’ inability to offer learning support to their children. This quote illustrates the problems:

For now, like I told you, because of the type of parents that we have. We even tried to create a WhatsApp page, but it was not successful because most of them are not on WhatsApp. So it has been difficult trying to give them online tuition, and looking at our situation, it is something that most of them cannot afford. I would say about maybe 85 to 90% cannot [afford to buy data and keep their WhatsApp running]. They will even tell you that they do not have the money to [buy mobile data and download assignments for their ward]. (SC3P3)

Notably, one school adopted an unconventional approach to encourage remote learning by placing textbooks and learning materials in front of the school premises for students to pick up. Students used the same approach to return assignments. In fact, of the nine schools sampled, only two had bespoke software with preloaded materials for teaching and learning as well as for communication with parents. The quote below is illustrative:

My school has a software package that enables us to send homework and classwork to parents. So we quickly decided to send our homework and assignments. With this, we have even finished tuition. We sent the assignment for about 2 weeks, then we stopped because technically we are supposed to be on vacation. So, if by the reopening of next term (May 5, 2020) we are still at home, then we will send the rest via the software. (SC1P1)

From the perspective of organizational ambidexterity, the transition to remote schooling and asynchronous learning constituted an ambidextrous strategy in that, although most schools, except SC1PI and SC2P2, did not have the modern ICT infrastructure and fast Internet connection system in place to deliver remote learning, they had to rely on existing assets, such as subject teachers, who were adept at using messaging software, such as WhatsApp, to send and receive assignments. It should also be mentioned that, in transitioning toward remote and online learning, most LFPSs and mid-range schools deliberately avoided strategies that required substantial financial commitments, such as investing in new ICT infrastructure, procuring specialized online schooling software packages, and paying teachers to prepare and deliver contents. As one proprietor disclosed, “We are not sure the parents will keep paying the fees if we invested in these modern virtual schooling software like Google Classroom and others” (SC9P9).

#### Changes to fee-collection strategies

Following the substantial amount of unrecovered tuition fees and some parents’ reticence to repay outstanding fees, nearly all the proprietors said they were either suspending the arrangements for installment payment altogether or increasing the proportion of down payments demanded from parents at the start of each term. They argued that, although the strategy seemed harsh and potentially at odds with their mission to broaden educational access to the poor, it was the only way they could survive, should similar unexpected events occur in the future. One proprietor illustrated this:

We have been badly hit because we gave people a lot of room to make an installment payment, but I am going to change that going forward. If we had collected all the fees before the pandemic struck, we would not have been in this situation. (SC1P1)

Again, from an ambidextrous standpoint, this response draws on both explorative and exploitative strategies. Indeed, as an exploitative strategy, although schools recognized there was a greater risk in continuing with the installment payments, some proprietors did not cancel the arrangement. Rather, they proposed to build on it by increasing the proportion of the initial down payment collected at the start of term. From an explorative standpoint, however, some schools charted an unknown path by canceling installment arrangements altogether. This is because of the palpable danger they would lose students from poor backgrounds, and it might take some time before students whose parents could make full payment at the start of term could be found as replacements.

#### Salary cuts and staff layoffs

Typically, most basic private schools have between 10 and 20 staff members, comprising teaching and non-teaching staff (Table 1). These staff members are mainly paid from the tuition fees collected (Inusah, 2020). To reduce cost burdens on schools at a time when no tuition fees were coming in, school proprietors indicated that they adopted two actions pertaining to their staff members. The first entailed laying off staff who did not play any role in the school-initiated remote learning exercise, as one proprietor said, “Unfortunately, we had to let some of the staff members who were not part of the online schooling go because no salaries were coming in and we couldn’t pay them” (SC9P9). Another said, “If we keep them on the payroll, then even if we don’t pay their full salaries, we still have to pay their SSNIT contributions, so we had to let them go” (SC3P3). The second strategy entailed reducing staff salaries, in some cases by as much as half the agreed salary. A proprietor testified, “The staff knew times were hard and so they agreed to take home half of their salary for April since parents had stopped paying tuition fees” (SC2P2).

It is important to emphasize that this decision was not taken lightly by proprietors, given the potential adverse impacts it would have on staff members, as one proprietor said, “I know it was a very difficult decision, but there was nothing we could do” (SC4P4). Again, both exploratory and exploitative strategies were deployed by proprietors in their responses to staff salary payment. The former related to laying off staff members without knowing how long the lockdown would last and recruiting new staff members later, while the exploitative strategy related to keeping some staff members adept at ICT to support online schooling.

#### Changes in use of existing assets

Given the uncertainty regarding when schools will reopen and bearing in mind that some schools used assets such as classroom blocks to organize vacation classes and shops for retailing stationary goods to generate extra income, some proprietors said they could not afford to allow their investments to remain underutilized. Consequently, some considered switching from running a school to other businesses that were allowed to operate during the lockdown, as one proprietor observed, “We have decided to close the school and give the premise to a cosmetology school. We will also use part of it for apartments” (SC7P7). Another disclosed that they would

Consider diversification so that there is multiple income flow. You know not all businesses have been affected by the lock down. For example, I have a personal rental service and for them, they pay in advance so you will have some money. (SC2P2)

From an organizational ambidextrous standpoint, these responses are predominantly exploitative in that they take advantage of existing school assets to maximize their use and generate extra income.

Some school proprietors who had excess food stuffs said they either distributed them among their neighbors or sold them at discounted prices out of fear that they might go bad and also to recoup some of their sunk capital (SC9P9, SC8P8).

## Conclusion

Following organizational ambidexterity (Lavie et al., 2010), we contend that the selected schools were able to respond to the impacts of the closure primarily due to two interrelated organizational factors: the type of school ownership model in place and the governance framework guiding decision-making in the school. In business management, a key advantage of sole proprietorship is that there is no clear separation between proprietors and their business, hence both profits and liabilities accrue to the person (Permwanichagun et al., 2016). Based on this premise, it appears that the actions taken by proprietors in response to unexpected closure including, for example, switching from running a school to another business, were predicated on the personal economic and social costs associated with losing the school, which is arguably the main investment and social security for the proprietors in their retirement. The implication of allowing personal economic and social circumstances to inform responses to closure is that, while the outcomes of the responses might guarantee the economic stability of the proprietors, the same cannot be said for the schools they own. This highlights the need for an intellectual discourse on epistemic questions regarding whether schools, particularly LFPSs, can be strictly considered businesses in the traditional sense.

Second, although the proprietors interviewed suggested that their schools had clear organizational structures, including a board of directors who set strategic visions, a school constitution that prescribed acceptable modes of conduct, division of labor to handle different tasks, and a clear chain of command in decision-making, it appears from the interviews that the sole proprietorship ownership model can override all these elements of good corporate governance. We observe that, although most of the proprietors interviewed repeatedly used the pronoun “we” when elaborating on their responses to the lockdown, no effort was made to suggest that decisions were subject to the approval of a school board or were an outcome of a consensus from the school management. Again, this emphasizes the need for an inquiry into the management of private schools and how they generally adhere to corporate governance systems in decision-making.

It is also worth emphasizing that most of the proprietors interviewed felt compelled to respond to the closure in the way they did because they lacked the “slack resources” (Voss et al., 2008), such as good ICT infrastructure systems and teachers with ICT training to support online schooling, to explore more interactive forms of remote learning. Furthermore, it is worth stressing that because most of the proprietors interviewed admitted to having received no formal academic qualification or training in educational leadership and school improvement, although many had overseen school management for over a decade and had gained invaluable on-the-job experience, the study makes clear that none of them had direct experience handling exogenous shocks until the pandemic. This may explain why some responses, such as canceling the installment payment system altogether, do not appear to have been informed by previous experience and a long-term strategic risk assessment of how their decision could indirectly exclude students from poor households in favor of their well-to-do counterparts.

The schools’ responses have implications for broadening educational access at the basic level. Indeed, it is well documented that private schools, particularly LFPSs, occupy an important place in the educational ecosystem of any country (Akaguri, 2011; Day Ashley et al., 2014). LFPSs also appear to provide a critical bridge for the relatively poor to ascend the social and economic ladder by receiving, arguably, quality education and earning a decent living subsequently (Inusah, 2020; Tooley, 2009). Nevertheless, our study shows that some private school responses, including canceling installment payments or increasing initial down payments and switching from operating a school to other businesses operations, imply that the poor in such schools are likely to be significantly negatively impacted. While this response appears to contradict the mission of LFPSs in terms of broadening access and improving educational quality for the poor, it has the unintended consequence of pushing the poor into public schools, which are often associated with low educational quality, especially in developing countries (Adelabu & Rose, 2004).

Our findings show that, contrary to the widespread notion that private schools are large profit-makers, hence they must be treated like all forms of well-capitalized business, some schools, such as LFPSs and mid-range private schools that allow flexible tuition-fee payment systems, hardly break even. Yet, they continue to be saddled with increasing statutory payments, without meaningful government support. For example, even with a government loan for small- to medium-scale enterprises (SMEs), proprietors interviewed said they had yet to benefit. Also, although the government supported all schools by providing them with noncontact thermometer guns and “veronica buckets” to aid handwashing, schools were excluded from government subsidies for water and electricity bill relief. Against this backdrop, it is imperative for the government to commission an inquiry into how many schools benefitted from the Covid-19 loans designed for SMEs, where such were located, their type of school, and how much each received. Findings from such an inquiry must be published so the public is informed. We also think the payment of statutory payments, such as SSNITs especially for staff members serving probation and property rates for LFPSs, deserve government attention, as they have proved to be stumbling blocks in private schools accessing government relief packages. In this regard, it might be worthwhile for the government to gather more data on such schools to properly design relief packages, including subsidized property rates and relaxed business operation permits.

From a theoretical standpoint, our work asks an important question related to whether the exploratory and exploitative strategies that businesses adopt in response to environmental crises, such as the Covid-19 school closures, must strictly inure to the benefit of the business per se or their shareholders or owners, and how this decision is made in the case of a sole proprietorship, for which it is difficult to clearly separate the owner’s burden from the costs and benefits to the organizations (LFPSs and mid-range private schools). Second, our study suggests that in analyzing the factors that allow organizations to adopt explorative, exploitative, or ambidextrous strategies, both organizational and management factors might not play out equally, and depending on the forms of business and the level of decision-making being analyzed, it is possible for either organizational or management factors to have differing impacts on the strategies adopted. Also, our findings seem to suggest that the dividing line between exploitative and explorative strategies is not clear cut, and these strategies are sometimes in flux, depending on how one look at them. This confirms Lavie’s (2010) position that perhaps it is best to view both strategies as the extreme ends of a continuum rather than disparate ends.
